# Extraction of Antioxidants from Borage (*Borago officinalis* L.) Leaves—Optimization by Response Surface Method and Application in Oil-in-Water Emulsions

**DOI:** 10.3390/antiox3020339

**Published:** 2014-05-06

**Authors:** Francisco Segovia, Bryshila Lupo, Sara Peiró, Michael H. Gordon, María Pilar Almajano

**Affiliations:** 1 Department of Chemical Engineering, Technical University of Catalonia, Avda. Diagonal 647, Barcelona 08028, Spain; E-Mails: segoviafj@gmail.com (F.S.); sapeisa@yahoo.es (S.P.); 2 Department of Chemical Engineering, Antonio José de Sucre National Experimental Polytechnic University, Avenida Corpahuaico, Barquisimeto 3001, Venezuela; 3 Department of Agro-industrial Engineering, Lisandro Alvarado Central Western University, Avenida Florencio Jiménez, Km 1, Barquisimeto 3001, Venezuela; E-Mail: bryshilalupo@ucla.edu.ve; 4 Department of Food and Nutritional Sciences, University of Reading, Whiteknights P.O. Box 226, Reading RG6 6AP, UK; E-Mail: m.h.gordon@reading.ac.uk

**Keywords:** RSM, rosmarinic acid, ORAC, borage leaves, extraction, emulsion, oxidation

## Abstract

Borage (*Borago officinalis* L.) is a typical Spanish plant. During processing, 60% are leaves. The aim of this work is to model and optimize the extraction of polyphenol from borage leaves using the response surface method (RSM) and to use this extract for application in emulsions. The responses were: total polyphenol content (TPC), antioxidant capacity by ORAC, and rosmarinic acid by HPLC. The ranges of the variables temperature, ethanol content and time were 50–90 °C, 0%–30%–60% ethanol (v/v), and 10–15 min. For ethanolic extraction, optimal conditions were at 75.9 °C, 52% ethanol and 14.8 min, yielding activity of 27.05 mg GAE/g DW TPC; 115.96 mg TE/g DW in ORAC and 11.02 mg/L rosmarinic acid. For water extraction, optimal activity was achieved with extraction at 98.3 °C and 22 min, with responses of 22.3 mg GAE/g DW TPC; 81.6 mg TE/g DW in ORAC and 3.9 mg/L rosmarinic acid. The significant variables were ethanol concentration and temperature. For emulsions, the peroxide value was inhibited by 60% for 3% extract concentration; and 80% with 3% extract concentration and 0.2% of BSA. The *p*-anisidine value between the control and the emulsion with 3% extract was reduced to 73.6% and with BSA 86.3%, and others concentrations had similar behavior.

## 1. Introduction

The properties of polyphenols as antioxidants have been widely recognized. They are associated with reduced risk of cancer, cardiovascular diseases, diabetes and Alzheimer’s disease [1]. Most polyphenols in the human diet are supplied by plants and fruits [2]. Furthermore, antioxidants from natural sources could be used to increase the stability of food, such as the ability to prevent lipid peroxidation [3]. This damage could be catalyzed by different metals present in food (especially in meat), because the metals can participate directly or indirectly in the reaction of oxidation of lipids [4]. In addition, these metals promote the creation of reactive oxygen species (ROS) prejudicial to health [5]. Polyphenols are also used as antimicrobial agents in food preservation [6].

The worldwide demand for food has been increasing. Nowadays, fresh fruit and vegetable production is, approximately, 800,000 t/year, without taking into account losses and waste [7]. In some studies polyphenols were found in pulp and other waste remaining from the production of fruit juices and wines [8,9]. Polyphenols can be excellent antioxidants and in some cases are better than synthetics ones [1]. New technology to treat food waste was required in order to obtain raw materials or ingredients for other processes and products [10].

Many health effects have been attributed to the borage (*Borago officinalis* L.) plant, such as: antispasmodic, antihypertensive, antipyretic, aphrodisiac, demulcent, and diuretic properties. It is also considered useful to treat asthma, bronchitis, cramps, diarrhea, palpitations, and kidney ailments [11]. In the food industry borage seed extracts have been used as effective antioxidants in the preparation of gelatin films from fish [12]. It was also shown to be effective in preventing oxidation in fermented dry sausages enriched with ω-3 polyunsaturated fatty acids (PUFA). As well as maintaining organoleptic properties, the borage extract was an economical and safe antioxidant source [1]. The antioxidant activity of borage meal extract was also demonstrated by Wettasinghe *et al.* (1999) [13] in a model meat system, where the inhibition of oxidation assessed by 2-thiobarbituric acid-reactive substances (TBARS), hexanal and total volatile formation was reported. Borage seed extracts exhibited strong metal chelating activity in an aqueous assay medium, that suggested it is a good chelating agent for food and non-food applications [4]. Bandoniene *et al.* (2002) [14] reported a study that showed that borage leaf extract was an effective antioxidant in rapeseed oil. The polyphenols found in borage include rosmarinic acid, which is responsible for some of the antioxidant properties of rosemary extracts, which is also widely used by the food industry. Rosmarinic acid has a high antioxidant capacity and it is present in the majority of Lamiaceae species [4,14,15,16].

Borage leaves are a cheap raw material for the production of polyphenols, because it is a by-product of an industrial process, and in addition, the disposal of this material incurs a cost, which can be minimized by its use [1].

Response surface methodology (RSM) is a useful tool for process optimization [17], that allows the influence of independent variables on a response variable to be represented by a mathematical model that is able to reproduce the behavior of these parameters, with only a few experiments [18,19]. An experimental design commonly used in the food industry is the central composite design (CCD), which involves evaluation of the factors at various levels [20].

Several foods such as: milk, sauces and soup have an emulsion structure. This could be oil in water (O/W) or water in oil (W/O) or a combination of both. Oxidation is a principal problem of this model [21]. The oxidation of emulsions differs from oil oxidation, due to the presence of oil or water droplets and an interface between oil and water, where components partition between the phases and interact with effects on chemical reactions [22]. Furthermore, in foods, there may be synergy between antioxidants and the protein present; which may increase the antioxidant capacity and enhance the stabilization of the emulsion [23,24].

In this work, we modeled and optimized the extraction of polyphenols from borage leaves based on the total polyphenols, antiradical activity (ORAC), and the amount of rosmarinic acid. The response surface method has not been used before, but it allowed the extraction parameters to be studied for optimization of antioxidant effects in a model emulsion system.

## 2. Experimental Section

### 2.1. Materials

2,2′-Azo-bis(2-amidinopropane) dihydrochloride (AAPH), was used as peroxyl radical source. Pyrogallol red (PGR), Trolox (6-hydroxy-2,5,8-tetramethylchroman-2-carboxylic acid), rosmarinic acid, ethanol, fluorescein, AAPH, BSA, *p*-anisidine (4-amino-anisole; 4-methoxy-aniline), isooctane, potassium persulfate, acetic acid (glacial) and polyoxyethylene sorbitan monolaurate (Tween-20) were purchased from Sigma-Aldrich Company Ltd. (Gillingham, UK). Folin–Ciocalteu reagent and sodium carbonate were supplied by Merck (Darmstadt, Germany). Refined sunflower oil, of a brand known to lack added antioxidants, was purchased from a local retail outlet. All compounds were of reagent grade.

### 2.2. Borage Preparation

The borage plant (*Borago officinalis* L.) was obtained in the local market, washed and the leaves were separated from other edible parts. This waste was homogenized and frozen at −80 °C for lyophilization. Then the leaves were ground into a powder by using a Moulinex mill (A5052HF, Moulinex, Lyon, France), then the particle size was standardized with a number 40 mesh sieve. Finally, the powder was stored in a dark bottle in a desiccator until use.

### 2.3. Extraction Procedure

Extraction was carried out in dark bottles, following the procedure described by Wijngaard *et al.* (2010) [8], with some slight modifications. Lyophilized sample powder (0.25 g) was blended with 15 mL of solvent of concentration specified by the CCD. It was mixed on a sample stirrer (SBS A-06 series H, Scientific Instrumentation SBS, Catalunya, Spain) for 1 min at 900 rpm, and then the liquid volume was increased to 25 mL with the solvent used. This mixture was placed in a bath by stirring at the required temperature and time specified by the experimental design, cooled in a refrigerator at 5 °C, centrifuged (Orto Alresa Mod. Consul, Ortoalresa, Ajalvir, Madrid, Spain) at 2500 rpm for 10 min, vacuum filtered and the loss solvent was replaced. The extract was stored at −20 °C until used for analysis.

### 2.4. Total Phenolic Content (TPC)

TPC was determined spectrophotometrically following the Folin–Ciocalteu colorimetric method [25]. Sample diluted 1:4 with milli-Q water was stirred in triplicate. The final concentration in the well (96 wells plate was used) was: 7.7% v/v sample, 4% v/v Folin-Ciocalteu’s reagent, 4% saturated sodium carbonate solution and 84, 3% of milli-Q water were mixed. The solution was allowed to react for 1 h in the dark and the absorbance was measured at 765 nm using a Fluorimetrics Fluostar Omega (BMG Labtech, Ortenberg, Germany). The total phenolic content was expressed as mg Gallic Acid Equivalents (GAE)/g dry weight.

### 2.5. ORAC Assay

Antioxidant activities of Borage extracts were determined by the ORAC assay, as reported by Ninfali *et al.* [26]. The assay was carried out using a Fluorimetrics Fluostar Omega (Perkin–Elmer, Paris, France) equipped with a temperature-controlled incubation chamber. The incubator temperature was set at 37 °C. The extract samples were diluted 1:20 with milli-Q water. The assay was performed as follows: 20% of sample was mixed with Fluorescein 0.01 mM, and an initial reading was taken with excitation wavelength, 485 nm and emission wavelength, 520 nm. Then, AAPH (0.3 M) was added measurements were continued for 2 h. This method includes the time and decrease of fluorescence. The area under the curve (AUC) was calculated. A calibration curve was made each time with the standard Trolox (500, 400, 250, 200, 100, 50 mM). The blank was 0.01 M phosphate buffered saline (pH 7.4). ORAC values were expressed as mg Trolox Equivalents (TE)/mg of dry borage.

### 2.6. HPLC

Identification and quantification of rosmarinic acid was performed using a Waters 2695 separations module (Meadows Instrumentation Inc., Bristol, WI, USA) system with a photodiode array detector Waters 996 (Meadows Instrumentation Inc., Bristol, WI, USA). The column was a Kinetex C18 100A, 100 × 4.6 mm (Phenomenex, Torrence, CA, USA). Solvents used for separation were 0.1% acetic acid in water (v/v) (eluent A) and 0.1% acetic acid in methanol (v/v) (eluent B). The gradient used was: 0–12 min, linear gradient from 40% to 50% B; 12–15 min, linear gradient from 50% to 40 B. The flow rate was 0.6 mL/min, and the detection wavelength was 330 nm. The sample injection volume was 10 μL. The chromatographic peak of rosmarinic acid was confirmed by comparing its retention time and diode array spectrum against that of a reference standard. Working standard solutions were injected into the HPLC and peak area responses obtained.

Standard graphs were prepared by plotting concentration (mg/L) *versus* area. Quantification was carried out from integrated peak areas of the samples using the corresponding standard graph.

### 2.7. Statistical Analysis

RSM was used to determine the optimal conditions of polyphenol extraction. A central composite design (CCD) was used to investigate the effects of three independent variables with two levels (solvent concentration, extraction temperature, and extraction time) with the dependent variables (TPC, ORAC activity, rosmarinic acid concentration). CCD uses the method of least-squares regression to fit the data to a quadratic model. The quadratic model for each response was as follows:

*Y* = *β_0_*+ ∑ *β_i_ X_i_* + ∑ *β_ii_ X_i_^2^* + ∑ ∑ *β_ij_ X_i_ X_j_* (1)

where, *Y* is the predicted response; 0 is a constant; *i* is the linear coefficient; *ii* is the quadratic coefficient, *ij* is the interaction coefficient of variables *i* and *j*; and *X_i_* and *X_j_* are independent variables.

The adequacy of the model was determined by evaluating the lack of fit, coefficient of determination (*R*^2^) obtained from the analysis of variance (ANOVA) that was generated by the software. Statistical significance of the model and model variables were determined at the 5% probability level (α = 0.05). The software uses the quadratic model equation shown above to build response surfaces. Three-dimensional response surface plots and contour plots were generated by keeping one response variable at its optimal level and plotting that against two factors (independent variables). Response surface plots were determined for each response variable. The coded values of the experimental factors and factor levels used in the response surface analysis are shown in Table 1. The graphics and the RSM analysis were made by software Matlab version R2013b (The MathWorks Inc., Natick, MA, USA).

**Table 1:** Design variable and code.

Extraction	Code	Temperature (°C)	Time (min)	Ethanol Concentration (%)
Ethanolic				
	−1	60	10	30
	0	70	15	45
	1	80	20	60
Aqueous				
	−1	50	10	
	0	70	15	
	1	90	20	

All responses were determined in triplicate and are expressed as average ± standard deviation. The answers have a percentage deviation less than 10%.

### 2.8. Oil-Water Emulsions

Oil-in-water emulsions (20.2 g) were prepared by dissolving Tween-20 (1%) in acetate buffer (0.1 M, pH 5.4), either with or without protein, namely BSA (0.2%), and borage extracts (3% v/v, 1% v/v, 0.3% v/v, 0.06% v/v). The emulsion was prepared by the dropwise addition of oil (sunflower oil) to the water phase, cooling in an ice bath with continuous sonication with a Vibracell sonicator (Sonics & Materials Inc., Newtown, CT, USA) for 5 min. All emulsions were stored in triplicate in 60 mL glass beakers in the dark (inside an oven) at 30 °C in an incubator. Two aliquots of each emulsion (0.005–0.1 g, depending on the extent of oxidation) were removed periodically for determination of peroxide value (PV) and *p*-anisidine value.

### 2.9. Peroxide Value (PV)

PV was determined by the ferric thiocyanate method (Frankel, 1998) [27] (after calibrating the procedure with a series of oxidized oil samples analyzed by the AOCS Official Method Cd 8-53). Data from the PV measurements were plotted against time.

### 2.10. p-Anisidine Value (p-AV)

The test was performed according to the methods reported by Singh *et al.* (2007) [28], with some modifications. In a 10 mL volumetric flask, 0.05 g of emulsion was taken and dissolved in 25% (v/v) of isooctane at 1% of acetic acid (glacial). From this solution, 2 mL was treated with 5% (v/v) of *p*-anisidine reagent and kept in the dark for 10 min and absorbance was measured at 350 nm using a UV-Vis spectrophotometer (Zuzi, AUXILAB, S.L., Beriain, Navarra, Spain).

## 3. Results and Discussion

### 3.1. Extraction

The extraction process was influenced by several factors including temperature, particle size, solvent, time, and solids characteristics. The polyphenols extraction was affected by increase of temperature and the solvent used [29]. Moreover, the effect of solvent-solid ratio was positive despite the solvents used, the higher solvent-solid ratio, and the higher total amount of solid obtained [30]. The total amount of polyphenols was increased by the reduction of particle size, however, with the smaller particle size results were less reproducible due to the formation of agglomerations of borage dry in samples [13]. On the other hand, the concentration used, optimal solvent and different processes promote the extraction of specific substances [10].

**Figure 1:**
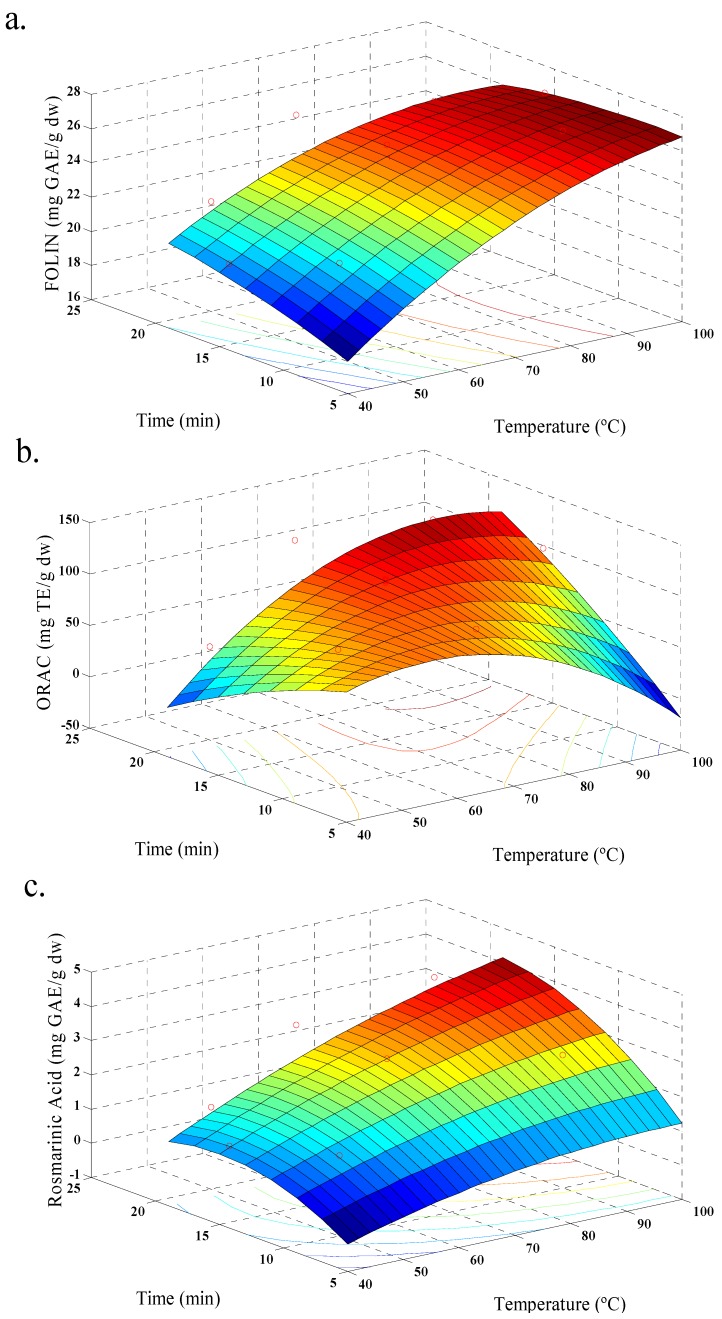
Response surface model plot, for aqueous extraction, showing the effects of time and temperature in: (**a**) total polyphenols contents; (**b**) antioxidant activity (ORAC); and (**c**) rosmarinic acid content.

**Figure 2:**
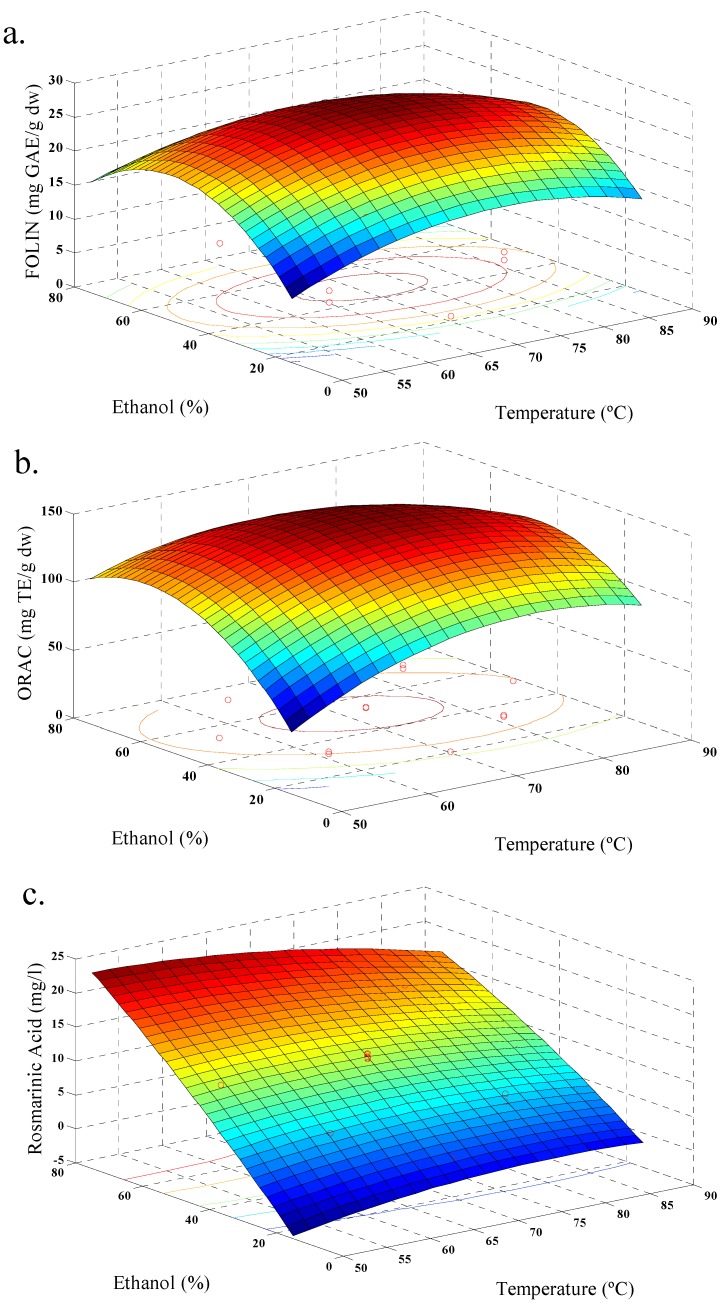
Response surface model plot, for ethanolic extraction, showing the effects of ethanol concentration and temperature on: (**a**) total polyphenols contents; (**b**) antioxidant activity (ORAC); and (**c**) rosmarinic acid content.

In our case, response values for each set of variable combinations for aqueous and ethanolic extraction were obtained (Table 2 and Table 3). All responses were adjusted to a quadratic model and the values of *R*^2^ were satisfactory (Table 4). Figure 1, shows the behavior of aqueous extraction, where, increasing temperature increases the amount of phenolic acid extracted, showing maximum values (Figure 1c). An increase of TPC and ORAC was observed too (Figure 1a,b). In addition, the yield of polyphenols was increased with increase in extraction time. This behavior is similar to that reported by Ballard *et al.* (2009) [19] in the extraction of peanut skin polyphenols. Figure 2, shows the behavior of the ethanolic extraction. It was similar to Figure 1, but with a decrease of TPC with increasing temperature (Figure 2a), and this behavior was also observed in previous studies of the effect of solvent polarity, temperature and time factors on ethanol extraction of defatted borage seed [31]. The effect may be explained by the degradation of some phenolic glycosides and flavonols at higher temperatures. The relationship between amount of polyphenols and antioxidant values were also observed for strawberry fruit extract [20]. The TPC values for both extractions reached a similar maximum value, although the conditions were different. In this sense, the extraction of polyphenols should be linked with solvent polarity and the extraction temperature [32], and for ethanolic extraction the optimal yield of polyphenols was occurred with the process conditions 70 °C, 45% of ethanol, and 15 min; while, for aqueous extraction the optimal polyphenol yield was obtained with conditions at 98.5 °C and 15 min.

**Table 2:** Experimental design and responses for aqueous extraction.

Temperature (°C)	Time (min)	TPC (mg GAE/g Dry Weight)	ORAC (mg TE/g Dry Weight)	Rosmarinic Acid (mg/L)
41.72	15.00	20.75 ± 0.58	27.22 ± 0.33	1.25 ± 0.01
70.00	15.00	24.89 ± 0.90	101.80 ± 6.37	2.79 ± 0.01
70.00	15.00	24.88 ± 0.87	104.05 ± 2.15	2.79 ± 0.01
90.00	10.00	26.45 ± 0.35	57.67 ± 2.76	2.87 ± 0.36
50.00	20.00	22.42 ± 0.93	40.36 ± 1.16	1.40 ± 0.01
70.00	7.93	24.16 ± 0.79	89.64 ± 1.55	1.52 ± 0.10
70.00	15.00	25.01± 0.07	103.24 ± 4.27	2.79 ± 0.01
50.00	10.00	21.58 ± 0.12	83.80 ± 2.21	1.36 ± 0.03
70.00	15.00	25.67 ± 0.55	104.87 ± 5.07	2.80 ± 0.01
70.00	15.00	24.49 ± 0.35	105.37 ± 1.15	2.80 ± 0.01
70.00	22.07	25.49± 0.54	110.92 ± 3.61	2.80 ± 0.21
90.00	20.00	25.46 ± 0.07	117.05 ± 1.88	3.79 ± 0.12
98.28	15.00	26.72 ± 0.79	102.23 ± 7.59	3.64 ± 0.04

GAE: Galic Acid Equivalent; TE: Trolox Equivalent.

The correspondence between the progressive increase of the rosmarinic acid concentration with the increase of antiradical capacity until reaching a maximum should be noted, due to the excellent antioxidant capacity of this component [15,33]. Moreover, rosmarinic acid was obtained in greater amounts by the ethanolic extraction, at 70% of ethanol solvent, where the yield was 5.6 times more than with the aqueous extraction. Other researchers have reported similar results when working with sage under the same conditions (Salvia oficinalis) [34]. Mhandi *et al.* (2007) [35] obtained an extract from borage seeds in which the amount of rosmarinic acid was similar to the maximum observed in the extractions carried out in this work. An equation that modeled the process of rosmarinic acid extraction was developed, and it was shown that rosmarinic acid yield decreased with the increase of ethanol in the solvent. The amount of rosmarinic acid obtained in the ethanolic extraction was higher than that obtained by aqueous extraction, and this behavior was observed in other studies [16]. Variation of conditions (temperature, ratio of solid/liquid) could not give good yields of rosmarinic acid when water was used as solvent, but other phenolic compounds may be efficiently extracted with water as observed in Figure 1b, where the ORAC values with water were close to those with ethanolic extraction. The ORAC values of the extract with ethanol were only 25% more than those obtained by aqueous extraction. Other studies [14,16] relate variability in the antiradical values to the actions of the factors mentioned earlier. Extracts of orange, apple, leek, and broccoli were investigated in other studies to determine the interactions [36].

**Table 3:** Experimental design and responses for ethanolic extraction.

Temperature (°C)	Ethanol Concentration (%)	Time (min)	TPC (mg GAE/g Dry Weight)	ORAC (mg TE/g Dry Weight)	Rosmarinic Acid (mg/L)
86.82	45.00	15.00	23.93 ± 1.06	126.80 ± 2.21	8.82 ± 0.13
60.00	30.00	10.00	20.26 ± 0.71	108.70 ± 1.23	3.77 ± 0.64
80.00	60.00	20.00	22.58 ± 1.23	125.21 ± 2.77	14.08 ± 0.07
70.00	45.00	15.00	27.49 ± 1.77	141.77 ± 3.19	11.04 ± 0.46
70.00	45.00	15.00	27.02 ± 1.04	146.06 ± 3.57	11.13 ± 0.57
70.00	45.00	15.00	26.91 ± 0.92	144.00 ± 6.42	11.43 ± 0.96
70.00	45.00	15.00	27.13 ± 1.15	143.72 ± 0.35	11.10 ± 0.58
70.00	45.00	22.07	25.33 ± 1.64	143.79 ± 3.40	11.83 ± 0.53
60.00	60.00	20.00	23.86 ± 0.69	128.13 ± 2.03	17.20 ± 1.15
60.00	30.00	20.00	22.43 ± 1.07	109.47 ± 3.88	5.38 ± 0.13
53.18	45.00	15.00	23.38 ± 0.83	124.97 ± 1.29	11.62 ± 0.54
70.00	19.77	15.00	19.16 ± 0.11	109.26 ± 3.34	0.77 ± 0.02
70.00	45.00	7.93	24.57 ± 0.54	138.27 ± 1.62	11.68 ± 0.07
70.00	45.00	15.00	27.16 ± 1.21	143.88 ± 3.05	11.23 ± 0.72
80.00	30.00	20.00	22.27 ± 0.72	128.78 ± 1.81	5.83 ± 0.28
60.00	60.00	10.00	22.99 ± 0.12	140.22 ± 0.57	17.53 ± 1.30
80.00	60.00	10.00	23.97 ± 1.37	146.88 ± 4.77	16.64 ± 0.47
70.00	45.00	15.00	26.94 ± 0.87	143.86 ± 0.20	11.30 ± 0.80
70.00	70.23	15.00	23.50 ± 0.76	132.04 ± 5.82	20.30 ± 0.00
80.00	30.00	10.00	23.25 ± 0.75	122.09 ± 2.23	4.69 ± 0.78

GAE: Gallic Acid Equivalent; TE: Trolox Equivalent.

**Table 4:** Mathematical equations from response surface method (RSM) for each of the responses, with their respective value of *R*^2^ and *R*^2^-predicted.

Extraction	Equation	*R*^2^ Value	
Response		*R* ^2^	*R*^2^-Pred.
*Ethanolic*			
TPC(mg GAE/g DW)	−132.03 + 2.37 *T* + 1.18 *C* + 3.13 *t* − 0.013 *T*^2^ − 0.009 *C*^2^ − 0.03 *t*^2^ − 0.003 *T* × *C* − 0.014 *T* × *t* − 0.003 *C* × *t*	97.8	84.8
ORAC(mg TE/g DW)	−544.88 + 11.18 *T* + 7.75 *C*+ 6.97 *t* − 0.068 *T*^2^ − 0.038 *C*^2^ − 0.058 *t*^2^ − 0.024 *T* × C − 0.009 *T* × *t* − 0.069 *C* × *t*	93.5	51.2
Rosmarinic Acid (mg/L)	−58.94 + 0.88 *T* + 1.09 *C* + 0.52 *t* − 0.004 *T*^2^ − 0.0012 *C*^2^ + 0.006 *t*^2^ − 0.004 *T* × *C* − 0.007 *T* × *t* − 0.009 *C* × *t*	99.7	97.6
*Aqueous*			
TPC(mg GAE/g DW)	−132.03 + 2.371 *T* + 1.18 *t* − 0.002 *T*^2^ − 0.006 *t*^2^ − 0.005 *T* × *t*	96.3	83.8
ORAC(mg TE/g DW)	−9.236 + 4.654 *T* − 12.357 *t* − 0.0538 *T*^2^ − 0.149 *t*^2^ − 0.257 *T* × *t*	94.5	61.1
Rosmarinic Acid (mg/L)	−67.250 + 1.233 *T* + 4.140 *t* − 0.009 *T*^2^ − 0.146 *t*^2^ − 0.005 *T* × *t*	98.5	89.1

*T*: Temperature (°C); *C*: Ethanol concentration (%); *t*: Time (min); Pred.: response predicted by model.

### 3.2. Response Surface Method

The RSM has been successfully applied to identify optimal conditions for the polyphenol extraction process, since it allows a mathematical model to be developed and through it the antioxidant values are linked with the factors such as temperature, solvent type, particle size, time, as well as others. In this sense, reported investigations have related the extraction of polyphenols and their antioxidant capacity from Castanea sativa leaves with time, temperature and solid/liquid ratio to identify the optimal conditions [37]. Pompeu *et al.* (2009) [18] varied the ethanol proportion in the solvent, composition and temperature to obtain an equation that described the process adequately. The antioxidant capacity of borage extract has been studied with respect to the factors: organic solvents, temperature and time; but the yield of a specific polyphenol has not been investigated [31]. In addition, no previous studies have been performed with water as solvent and the effect of variation of extraction temperature and yield of rosmarinic acid have not been reported. The use of water as solvent has the advantages that it is environmentally safe and cheap [38].

Table 5 shows the *p* values for each of the coefficients of the quadratic terms obtained by using RSM. In extractions with variable time and temperature (for ORAC) and ethanol (for TPC), moreover the quadratic term (for ORAC and rosmarinic acid), had high values of *p* (*p* > 0.05). The high *p* values were attributed to fast mass transfer of phenolic compounds, which was dependent on the manner in which the sample was processed (powder) so that the quadratic term may or may not be significant [39,40]. The most significant variable was the concentration of ethanol in the ethanolic extraction, which had an influence on all responses. Similarly, for aqueous extraction the *p* values were high for terms including the time factor for TPC and ORAC. In addition, the constant was not significant for the model. However, for the yield of rosmarinic acid the equation coefficients were highly significant, which shows that the mathematics model may be representing the extraction adequately.

### 3.3. Validation Conditions Optimized

Optimal conditions obtained for each of the extractions and for each type of extraction were carried out in triplicate and the responses were obtained (Table 6). For predicting responses, the model found fits well with the actual values, except in the case of the ORAC values, where it showed a deviation from the expected values based on the value of *R*^2^ predictive. Moreover, there was a difference between extractions. The ethanolic extraction gave higher values in: TPC, ORAC, and rosmarinic acid than the aqueous extraction.

### 3.4. Oil-Water Emulsions

The progress of oxidation with time in the stored emulsion samples was measured by the PV and *p*-anisidine methods, which have been widely used for this purpose [41,42]. The total period of the experiment was limited to 864 h, because at that moment the stability of the emulsion was broken since it separated into separate phases. The extract used was obtained by ethanolic extraction with the optimal conditions found earlier, since the extract has maximum values for the ORAC value and rosmarinic acid content.

**Table 5:** *p*-Values for each of the constants in the equation of the mathematical model.

Extraction	Term	*p* Value		
		Response		
		TPC	ORAC	Rosmarinic Acid
Ethanolic				
	Constant	0.000	0.001	0.000
	Temperature (°C)	0.000	0.000	0.000
	Ethanol (%)	0.000	0.000	0.000
	Time (min)	0.000	0.092	0.133
	Temperature (°C) × Temperature (°C)	0.000	0.000	0.003
	Ethanol (%) × Ethanol (%)	0.000	0.000	0.022
	Time (min) × Time (min)	0.000	0.248	0.149
	Temperature (°C) × Ethanol (%)	0.044	0.046	0.001
	Temperature (°C) × Time (min)	0.003	0.780	0.031
	Ethanol (%) × Time (min)	0.236	0.009	0.000
Aqueous				
	Constant	0.536	0.899	0.000
	Temperature (°C)	0.001	0.011	0.000
	Time (min)	0.077	0.045	0.000
	Temperature (°C) × Temperature (°C)	0.005	0.000	0.000
	Time (min) × Time (min)	0.400	0.299	0.000
	Temperature (°C) × Time (min)	0.088	0.001	0.180

TPC in mg GAE/g DW; ORAC in mg TE/g DW; Rosmarinic Acid in mg/L. GAE: Gallic Acid Equivalent; TE: trolox equivalent.

**Table 6:** The optimal responses given by RSM for the two types of extractions.

Extraction	Conditions			Response					
	Temperature (°C)	Ethanol (%)	Time (min)	TPC		ORAC		Rosmarinic Acid	
				Predicted	Actual	Predicted	Actual	Predicted	Actual
Ethanolic	75.94	51.88	14.8	26.71	27.05	145.03	115.96	13.29	11.024
Aqueous	98.28	-	22.07	26.02	22.27	120.33	81.6	4.09	3.9

GAE: Gallic Acid Equivalent; TE: trolox equivalent; TPC in mg GAE/g DW; ORAC in mg TE/g DW; Rosmarinic Acid in mg/L.

**Figure 3:**
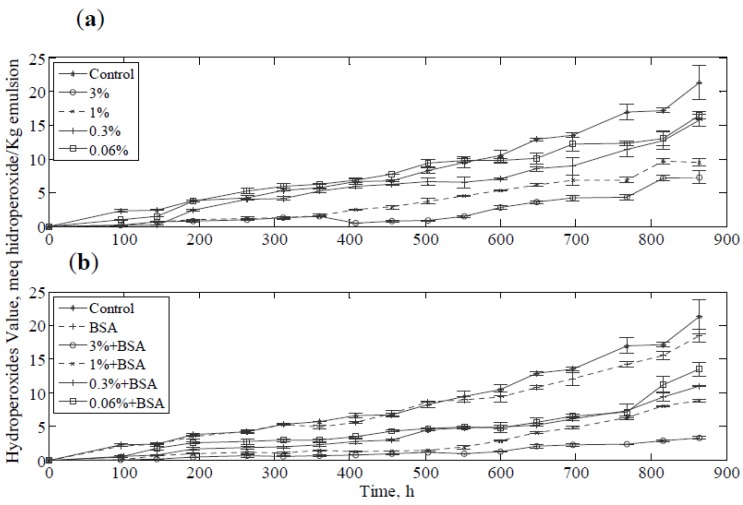
The peroxide values for the emulsions: (**a**) Samples with extract; (**b**) Samples with extract and 0.2% BSA.

Figure 3 shows the PV of the emulsion with different concentration of borage extract. In Figure 3a, a clearly increase in inhibition of lipid oxidation by increasing the extract concentration was observed and the reduction in PV suggested an effect of protection for the oil phase. This effect of borage extracts was indicated by other authors, who demonstrated the influence of the concentration of polyphenol extracts of different plants in the oxidative process in the oil [43] and they also reported the dependence of the antioxidant activity on the type of polyphenol. Polar polyphenols were more soluble in the aqueous phase and less polar polyphenols were concentrated at the interface between oil-water [44]. In this sense, rosmarinic acid may be the cause of antioxidant activity in the aqueous phase as found by Jordán *et al.* (2012) [16]. The emulsions containing extract in the range of concentrations from 0.06% to 3% had an antioxidant capacity between 8% and 60% considering the control emulsion as reference. Moreover, the emulsions containing 1% and 3% of extract presented a significant difference (*p* < 0.05) in their PV with respect to the control sample.

BSA has been shown to increase antioxidant activity by synergy with phenolic antioxidants in emulsions. As in other studies, stabilization of the emulsion was observed by PV measurements [45]. The negative character of the protein and the positive charge of metal ions could explain this increase in the antioxidant capacity [21]. Furthermore, the protein contributes to physical stabilization of the emulsion and unabsorbed protein decreases lipid oxidation [46]. Figure 3b shows the PV values of the stored emulsion containing extract and BSA. It was evident that the BSA contributed to the antioxidant activity in the emulsion system, and the emulsion was significantly more stable when assessed by the PV than the sample without protein. In fact, the emulsions containing 0.2% of BSA and extract in the range 0.06% to 3% had an antioxidant capacity between 36.6% and 84% considering the control emulsion as reference. These results indicated for all concentrations employed samples containing extract and BSA were significantly more stable (*p* < 0.05) than the control emulsion with and without BSA.

**Figure 4:**
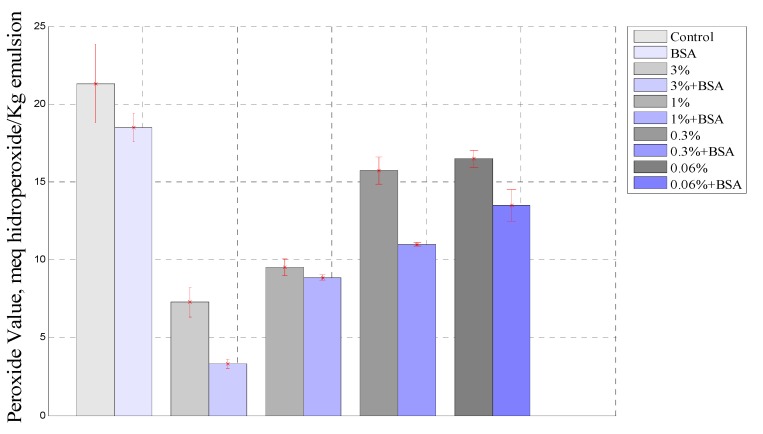
Comparison between samples containing extract and extract plus BSA at 864 h.

Figure 4 show a comparison of PV behavior between all emulsions studied at the end of the experiment after 864 h. As was observed, the PV in the emulsion with borage extract was higher than that for the emulsion containing borage extract and BSA, and it was noted that the BSA greatly improved the antioxidant effect of the extract. The stability of the emulsion play an important role in the oxidation process, since the protein enhances droplet formation and forms a layer thereby protecting the oil droplet [47]. Moreover, it was observed that the sample containing BSA had a lower PV than the control emulsion.

**Figure 5:**
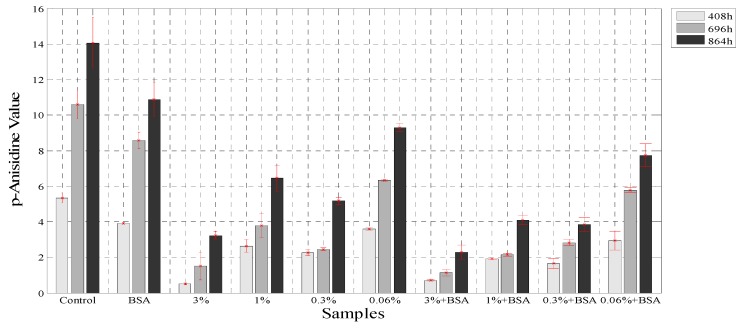
The *p*-anisidine values for the samples with BSA and without BSA.

The secondary products of oxidation in emulsion were measured with the *p*-anasidine value test, and this method has been used for the determination of antioxidant capacities of different oils [28], emulsions [22] and the effects of extracts in oils [48]. The *p*-anisidine values (*p*-AV) were evaluated at three times: 408 h, 696 h, and 864 h with the same samples used for PV analysis. The determination of *p*-anisidine values has been used to show a synergy for the combination of both, BSA and polyphenol extract obtained from other plants like green tea [23]. Figure 5 shows that the borage extract at 3%, in combination with BSA, reduced by 86.3% the formation of secondary oxidation products in the samples, whereas the extract at 3% reduced the *p*-anisidine values by 73.6% at 864 h. However, the *p*-AV increased throughout the experiment. The presence of protein at the interface decreased the rate of lipid oxidation due to its ability to trap free radicals and bind with metals as seen earlier [49]. In addition, studies carried out with green tea extracts and emulsion samples showed an inhibitory effect on *p*-AV similar to that in the current study, since the hydrophilic catechins present in the green tea extract acted at the oil-water interface like rosmarinic acid and reduced the rate of the oxidation process [50].

## 4. Conclusions

A model has been developed to describe the effect of several variables on extraction of polyphenols from borage leaves. The antioxidant activity (ORAC value) demonstrated optimal values for ethanolic and for aqueous extraction. The increase in antioxidant capacity can be related to the increase of the amount of rosmarinic acid; however other polyphenols may also contribute, as seen by the decrease of ORAC values with increase of extraction temperature. The decomposition of these polyphenols may explain these results. The ethanolic or aqueous extraction conditions can be chosen according to the type of phenolic acids we want to enrich in the extract, or the effect we want to obtain. The use of water for extraction of polyphenols made the extraction process more environmentally friendly, but increased the energy used. The time variable was of little significance for the model.

The use of the borage extract and the extract in combination with BSA decreased the rate of increase of PV and *p*-AV in an emulsion, with a synergistic effect demonstrated. This effect could be associated with the presence of rosmarinic acid in the extract and the function of BSA as a metal chelating agent at the interface.

The application of innovative technologies such as ultrasound, electromagnetic pulses, subcritical water extraction, among other, could be applied for further study in order to enhance the extraction of polyphenols [38,51,52] of the borage leaves, as well as its influence on preservation of the antioxidant properties on the extract obtained.

## References

[1] Krishnaiah D., Sarbatly R., Nithyanandam R. (2011). A review of the antioxidant potential of medicinal plant species. Food Bioprod. Process..

[2] Manach C., Scalbert A., Morand C., Rémésy C., Jiménez L. (2004). Polyphenols: food sources and bioavailability. Am. J. Clin. Nutr..

[3] Gironi F., Piemonte V. (2011). Temperature and solvent effects on polyphenol extraction process from chestnut tree wood. Chem. Eng. Res. Des..

[4] Wettasinghe M., Shahidi F. (2001). Phenolic acids in defatted seeds of borage (*Borago officinalis* L.). Food Chem..

[5] Finley J.W., Kong A.-N., Hintze K.J., Jeffery E.H., Ji L.L., Lei X.G. (2011). Antioxidants in foods: State of the science important to the food industry. J. Agric. Food Chem..

[6] Perumalla A.V.S., Hettiarachchy N.S. (2011). Green tea and grape seed extracts—Potential applications in food safety and quality. Food Res. Int..

[7] Ayala-Zavala J.F., Vega-Vega V., Rosas-Domínguez C., Palafox-Carlos H., Villa-Rodriguez J.A., Siddiqui M.W., Dávila-Aviña J.E., González-Aguilar G.A. (2011). Agro-industrial potential of exotic fruit byproducts as a source of food additives. Food Res. Int..

[8] Wijngaard H., Brunton N. (2010). The optimisation of solid-liquid extraction of antioxidants from apple pomace by response surface methodology. J. Food Eng..

[9] Guerrero M.S., Torres J.S., Nuñez M.J. (2008). Extraction of polyphenols from white distilled grape pomace: Optimization and modelling. Bioresour. Technol..

[10] Wijngaard H., Brunton N. (2009). The optimization of extraction of antioxidants from apple pomace by pressurized liquids. J. Agric. Food Chem..

[11] Gilani A.H., Bashir S., Khan A. (2007). Pharmacological basis for the use of *Borago officinalis* in gastrointestinal, respiratory and cardiovascular disorders. J. Ethnopharmacol..

[12] Gómez-Estaca J., Montero P., Giménez B., Gómez-Guillén M.C. (2009). Incorporation of antioxidant borage extract into edible films based on sole skin gelatin or a commercial fish gelatin. J. Food Eng..

[13] Bucić-Kojić A., Planinić M., Tomas S., Bilić M., Velić D. (2007). Study of solid-liquid extraction kinetics of total polyphenols from grape seeds. J. Food Eng..

[14] Bandoniene D. (2002). The detection of radical scavenging compounds in crude extract of borage (*Borago officinalis* L.) by using an on-line HPLC-DPPH method. J. Biochem. Biophys. Methods.

[15] Wang H., Provan G.J., Helliwell K. (2004). Food chemistry determination of rosmarinic acid and caffeic acid in aromatic herbs by HPLC. Food Chem..

[16] Jordán M., Lax V., Rota M.C., Lora S., Sotomayor J.A. (2012). Relevance of carnosic acid, carnosol, and rosmarinic acid concentrations in the *in vitro* antioxidant and antimicrobial activities of (*Rosmarinus officinalis* L.) methanolic extracts. J. Agric. Food Chem..

[17] Sun Y., Xu W., Zhang W., Hu Q., Zeng X. (2011). Optimizing the extraction of phenolic antioxidants from kudingcha made from *Ilex kudingcha* C.J. Tseng by using response surface methodology. Sep. Purif. Technol..

[18] Pompeu D.R., Silva E.M., Rogez H. (2009). Optimisation of the solvent extraction of phenolic antioxidants from fruits of *Euterpe oleracea* using Response Surface Methodology. Bioresour. Technol..

[19] Ballard T.S., Mallikarjunan P., Zhou K., O’Keefe S.F. (2009). Optimizing the extraction of phenolic antioxidants from peanut skins using response surface methodology. J. Agric. Food Chem..

[20] Saha J., Debnath M., Saha A., Ghosh T., Sarkar P.K. (2011). Response surface optimisation of extraction of antioxidants from strawberry fruit, and lipid peroxidation inhibitory potential of the fruit extract in cooked chicken patties. J. Sci. Food Agric..

[21] Cheng Y., Xiong Y.L., Chen J. (2010). Antioxidant and emulsifying properties of potato protein hydrolysate in soybean oil-in-water emulsions. Food Chem..

[22] Dwyer S.P., Beirne D., Ní D., Kennedy B.T. (2013). Effects of sodium caseinate concentration and storage conditions on the oxidative stability of oil-in-water emulsions. Food Chem..

[23] Almajano M.P., Delgado M.E., Gordon M.H. (2007). Albumin causes a synergistic increase in the antioxidant activity of green tea catechins in oil-in-water emulsions. Food Chem..

[24] Elias R.J., Mcclements D.J., Decker E.A. (2007). Impact of thermal processing on the antioxidant mechanisms of continuous phase β-lactoglobulin in oil-in-water emulsions. Food Chem..

[25] Singleton V., Rossi J. (1965). Colorimetry of total phenolics with phosphomolybdic-phosphotungstic acid reagents. Am. J. Enol. Vitic..

[26] Ninfali P., Mea G., Giorgini S., Rocchi M., Bacchiocca M. (2007). Antioxidant capacity of vegetables, spices and dressings relevant to nutrition. Br. J. Nutr..

[27] Frankel E.N. (1998). Methods to Determine Extent of Oxidation. Lipid Oxidation.

[28] Singh G., Maurya S., deLampasona P., Catalan C. (2007). A comparison of chemical, antioxidant and antimicrobial studies of cinnamon leaf and bark volatile oils, oleoresins and their constituent. Food Chem. Toxicol..

[29] Cacace J.E., Mazza G. (2003). Mass transfer process during extraction of phenolic compounds from milled berries. J. Food Eng..

[30] Pinelo M., Rubilar M., Jerez M., Sineiro J., Núñez M.J. (2005). Effect of solvent, temperature, and solvent-to-solid ratio on the total phenolic content and antiradical activity of extracts from different components of grape pomace. J. Agric. Food Chem..

[31] Wettasinghe M., Shahidi F. (1999). Antioxidant and free radical-scavenging properties of ethanolic extracts of defatted borage (*Borago o cinalis* L.) seeds. Food Chem..

[32] Naczk M., Shahidi F. (2004). Extraction and analysis of phenolics in food. J. Chromatogr. A.

[33] Rahimi A., Hashemi P., Badiei A., Safdarian M., Rashidipour M. (2013). Microextraction of rosmarinic acid using CMK-3 nanoporous carbon in a packed syringe. Chromatographia.

[34] Durling N., Catchpole O., Grey J., Webby R., Mitchell K., Foo L., Perry N. (2007). Extraction of phenolics and essential oil from dried sage (*Salvia officinalis*) using ethanol-water mixtures. Food Chem..

[35] Mhamdi B., Wannes W.A., Bourgou S., Marzouk B. (2009). Biochemical characterization of borage. J. Food Biochem..

[36] Michiels J.A., Kevers C., Pincemail J., Defraigne J.O., Dommes J. (2012). Extraction conditions can greatly influence antioxidant capacity assays in plant food matrices. Food Chem..

[37] Díaz Reinoso B., Couto D., Moure A., Fernandes E., Domínguez H., Parajó J.C. (2012). Optimization of antioxidants—Extraction from *Castanea sativa* leaves. Chem. Eng. J..

[38] Fernández-Ponce M.T., Casas L., Mantell C., Rodríguez M., Martínez de la Ossa E. (2012). Extraction of antioxidant compounds from different varieties of *Mangifera indica* leaves using green technologies. J. Supercrit. Fluids.

[39] Pinelo M., Sineiro J., Núñez M.J. (2006). Mass transfer during continuous solid-liquid extraction of antioxidants from grape byproducts. J. Food Eng..

[40] Fiori L., Basso D., Costa P. (2008). Seed oil supercritical extraction: Particle size distribution of the milled seeds and modeling. J. Supercrit. Fluids.

[41] Owczarek-Fendor A., Meulenaer B., Scholl G., Adams A., van Lancker F., Yogendrarajah P., Uytterhoeven V., Eppe G., de Pauw E., Scippo M.-L. (2010). Importance of fat oxidation in starch-based emulsions in the generation of the process contaminant furan. J. Agric. Food Chem..

[42] Poyato C., Navarro-blasco I., Isabel M., Yolanda R., Astiasarán I., Ansorena D. (2013). Oxidative stability of O/W and W/O/W emulsions: Effect of lipid composition and antioxidant polarity. Food Res. Int..

[43] Wardhani D.H., Fuciños P., Vázquez J.A., Pandiella S.S. (2013). Inhibition kinetics of lipid oxidation of model foods by using antioxidant extract of fermented soybeans. Food Chem..

[44] Ramful D., Aumjaud B., Neergheen V.S., Soobrattee M.A., Googoolye K., Aruoma O.I., Bahorun T. (2011). Polyphenolic content and antioxidant activity of *Eugenia pollicina* leaf extract in vitro and *in model* emulsion systems. Food Res. Int..

[45] Bonoli-Carbognin M., Erretani L.O.C., Endini A.L.B., Goidanich P., Cesena I. (2008). Bovine serum albumin produces a synergistic increase in the antioxidant activity of virgin olive oil phenolic compounds in oil-in-water emulsions. J. Agric. Food Chem..

[46] Ries D., Ye A., Haisman D., Singh H. (2010). Antioxidant properties of caseins and whey proteins in model oil-in-water emulsions. Int. Dairy J..

[47] Sun C., Gunasekaran S. (2009). Effects of protein concentration and oil-phase volume fraction on the stability and rheology of menhaden oil-in-water emulsions stabilized by whey protein isolate with xanthan gum. Food Hydrocoll..

[48] Abdelazim A.A., Mahmoud A. (2013). Oxidative stability of vegetable oils as affected by sesame extracts during accelerated oxidative storage. J. Food Sci. Technol..

[49] Kargar M., Spyropoulos F., Norton I.T. (2011). The effect of interfacial microstructure on the lipid oxidation stability of oil-in-water emulsions. J. Colloid Interface Sci..

[50] Dwyer S.P., Beirne D., Deirdre N., Kennedy B.T. (2012). Effects of green tea extract and α-tocopherol on the lipid oxidation rate of omega-3 oils, incorporated into table spreads, prepared using multiple emulsion technology. J. Food Sci..

[51] Luengo E., Álvarez I., Raso J. (2013). Improving the pressing extraction of polyphenols of orange peel by pulsed electric fields. Innov. Food Sci. Emerg. Technol..

[52] Chemat F., Zill-e-Huma, Khan M.K. (2011). Applications of ultrasound in food technology: Processing, preservation and extraction. Ultrason. Sonochem..

